# The AGEs/RAGE Axis Contributes to Myocardial Cell Death in Type 1 Diabetic Cardiomyopathy via the ROS/NF‐*κ*B/NLRP3 Inflammasome Pathway

**DOI:** 10.1155/jdr/6438008

**Published:** 2026-10-23

**Authors:** Xiaoqun Li, Shujie Chen, Ke Wang, Ke Xu, Yannan Wang, Zechao Zhang, Xue Liu

**Affiliations:** ^1^ State-Province Key Laboratories of Biomedicine-Pharmaceutics of China Key Laboratory of Cardiovascular Medicine Research Ministry of Education Department of Pharmacology College of Pharmacy Harbin Medical University Harbin China hrbmu.edu.cn

**Keywords:** advanced glycation end products (AGEs), diabetic cardiomyopathy, NLRP3 inflammasome, Type 1 diabetes mellitus

## Abstract

**Background:**

Type 1 diabetes mellitus (T1DM) cardiomyopathy is a severe complication that typically follows an insidious onset and then progresses rapidly to heart failure and premature death. AGEs accumulate in the myocardium under hyperglycemic conditions and have emerged as important mediators of this diabetic complication, although the full signaling cascades engaged by AGEs remain largely unresolved. In the present study, we therefore investigated whether AGEs induce myocardial injury in T1DM via NLRP3 inflammasome activation.

**Methods:**

A T1DM mouse model was established using streptozotocin (STZ) injections, and an in vitro model was generated by treating primary neonatal mouse cardiomyocytes with AGEs. Cardiac performance and morphological changes were examined via echocardiography and histopathological staining. Expression and activation of the NLRP3 signaling cascade were evaluated by Western blotting, ELISA, and immunofluorescence. Pyroptotic cell death was assessed by SYTOX green staining, PI uptake, and lactate dehydrogenase (LDH) release assay. Correlations between whole‐blood RAGE gene expression and inflammatory cytokine levels (IL‐1*β* and IL‐18) in T1DM patients, along with myocardial abundance of RAGE, NLRP3, and N‐GSDMD in the experimental mice, were analyzed as exploratory descriptive observations.

**Results:**

STZ‐induced T1DM mice exhibited sustained hyperglycemia (fasting blood glucose ≥ 11.1 mmol/L) and impaired cardiac function. Myocardial tissue from T1DM animals showed marked engagement of the NLRP3 signaling cascade, as evidenced by elevated NLRP3, cleaved caspase‐1 P20, and N‐GSDMD, enhanced ASC speck formation, and increased serum IL‐1*β* levels. In vitro, AGEs treatment directly triggered the NLRP3 pathway and drove cardiomyocyte pyroptosis. Mechanistically, AGEs binding to RAGE triggered a burst of intracellular ROS, which led to NF‐*κ*B activation and subsequent NLRP3 activation. These effects were suppressed by either the RAGE inhibitor FPS‐ZM1 or the ROS scavenger N‐acetylcysteine (NAC). Furthermore, MCC950 treatment significantly attenuated AGE‐induced caspase‐1 activation, IL‐1*β*/IL‐18 secretion, and N‐GSDMD expression, confirming that NLRP3 is a necessary mediator of this signaling cascade. SYTOX green staining, PI uptake, and LDH release assays further demonstrated that AGE‐induced pyroptotic cell death was blocked by FPS‐ZM1, NAC, and MCC950, indicating that pyroptosis occurs specifically through the RAGE/ROS/NLRP3 axis. Correlation analyses revealed that RAGE transcript levels in whole blood from T1DM patients positively correlated with circulating IL‐1*β* and IL‐18. In the diabetic mouse heart, RAGE, NLRP3, and N‐GSDMD protein levels were analyzed as exploratory descriptive observations and showed a consistent pattern of coexpression at the individual level.

**Conclusion:**

AGEs promote NLRP3 inflammasome activation in cardiomyocytes via the RAGE/ROS/NF‐*κ*B signaling axis, driving inflammatory responses and pyroptosis, thereby contributing to the progression of T1DM cardiomyopathy. These observations imply that modulating this signaling axis could serve as a promising therapeutic strategy for treating T1DM cardiomyopathy.

## 1. Introduction

The global burden of diabetes is escalating. Recent data from The Lancet show that the worldwide prevalence of diabetes has risen steadily from 1990 to 2021, with projections exceeding 1.31 billion cases by 2050. Although Type 1 diabetes mellitus (T1DM) accounts for only ~4% of all diabetes cases, it confers markedly elevated all‐cause and cardiovascular mortality relative to the general population [1, 2]. Unlike Type 2 diabetes mellitus (T2DM), which is frequently accompanied by metabolic syndrome, cardiovascular complications in T1DM often develop insidiously and progress gradually. In early stages, patients may lack traditional risk factors such as hypertension or dyslipidemia, which allows myocardial injury to progress silently and thus contribute to an overall poor prognosis [2–5]. Despite recent improvements in cardiovascular outcomes among T1DM patients [6], those with poor glycemic control still have an almost 10‐fold greater likelihood of cardiovascular incidents than nondiabetic individuals [3, 5]. Notably, conventional risk factors account for less than half of the excess cardiovascular burden seen in T1DM [4, 7], implying that disease‐specific pathological mechanisms are predominant. These mechanisms likely include the accumulation of advanced glycation end products (AGEs) due to chronic hyperglycemia and immune‐mediated chronic inflammation [8]. Clinical studies further suggest that the cardiovascular risk in T1DM may exceed that observed in T2DM [8], this notion is corroborated by animal models demonstrating more severe systolic and diastolic dysfunction of the left ventricle, along with more pronounced pathological myocardial remodeling, in T1DM compared with T2DM [9]. Thus, unraveling the molecular basis of myocardial damage in T1DM, particularly the inflammatory signaling pathways activated by AGEs, is crucial for understanding the pathophysiology of its cardiovascular complications.

The pathogenesis of T1DM cardiomyopathy is initiated by absolute insulin deficiency, resulting in persistent hyperglycemia. This pathological state accelerates the formation of AGEs and activates their receptor signaling pathways, which in turn induces myocardial inflammatory responses, oxidative stress injury, and potentially cardiac‐specific autoimmune reactions [10–12]. In contrast, T2DM cardiomyopathy is primarily driven by insulin resistance, often accompanied by lipotoxicity from disordered glucose‐lipid metabolism and activation of the RAAS, without significant involvement of autoimmune mechanisms [13, 14]. These distinctions suggest AGEs as pivotal mediators in T1DM‐related myocardial injury, whereas myocardial injury in T2DM relies more on the synergistic effects of hyperglycemia and dyslipidemia. These fundamental differences in their pathogenic mechanisms suggest that T1DM cardiomyopathy may involve unique therapeutic targets that warrant further investigation. Within the complex pathogenesis of T1DM cardiomyopathy, sustained hyperglycemia‐driven AGEs accumulation represents a critical step. AGEs are stable covalent compounds generated by nonenzymatic glycation involving reducing sugars and free amino groups found on proteins or lipids. Although this process occurs slowly under physiological conditions, it is markedly accelerated by the persistent hyperglycemia in T1DM, leading to abnormal AGEs deposition in myocardial tissue [15, 16]. These end products predominantly trigger key proinflammatory cascades such as NF‐*κ*B and MAPK through engagement of their specific receptor, RAGE, initiating downstream signaling events that induce oxidative stress cascades [17]. The ensuing inflammatory and oxidative damage mediated by the AGEs/RAGE axis contributes to cardiomyocyte death, dysregulated autophagy, and extracellular matrix remodeling, collectively driving the characteristic structural and functional decline of T1DM cardiomyopathy. A detailed understanding of the specific signaling networks controlled by AGEs in the T1DM heart is therefore essential for elucidating the unique disease progression and informing targeted therapeutic strategies.

The NOD‐like receptor protein 3 (NLRP3) inflammasome is a central component of innate immunity. When triggered by diverse pathogen‐ or damage‐associated molecular patterns, it promotes the maturation and secretion of interleukin‐1*β* (IL‐1*β*) and interleukin‐18 (IL‐18) in a caspase‐1–dependent manner, thereby initiating and amplifying inflammatory cascades. Excessive activation of the NLRP3 inflammasome is known to exacerbate myocardial inflammation, fibrosis, and functional impairment across various cardiovascular pathologies, including myocardial ischemia, heart failure, and diabetic cardiomyopathy [18–21]. In cardiomyocytes, the ROS‐driven NF‐*κ*B/NLRP3 signaling axis has been shown to contribute to pressure overload‐induced heart failure [21]. Evidences indicate that high glucose levels can stimulate the NLRP3 inflammasome activation [22–24]. However, although high glucose represents the initial metabolic disturbance in T1DM, it is the subsequent accumulation of AGEs—the stable end products of nonenzymatic glycation—that more directly mediates the chronic inflammatory injury in the diabetic heart. Indeed, AGEs have been identified as potent NLRP3 activators across multiple cell types, including corneal epithelial cells [25], renal podocytes [26], and neurons [27]. Nevertheless, whether AGEs directly regulate NLRP3 inflammasome activation in cardiomyocytes via RAGE in the context of T1DM cardiomyopathy has not been systematically investigated. Thus, clarifying the upstream regulatory function of the AGEs–RAGE cascade on the NLRP3 inflammasome is critical for deepening our understanding of T1DM cardiomyopathy.

Building on this foundation, the present study sought to examine the contribution of the AGEs/RAGE signaling axis in NLRP3 inflammasome activation in the pathogenesis of T1DM cardiomyopathy. We hypothesized that AGEs accumulation in cardiomyocytes promotes myocardial injury by triggering the NLRP3 inflammasome along with its downstream inflammatory pathways via engagement of RAGE. To test this hypothesis, we combined in vitro experiments using AGEs‐treated cardiomyocytes with interventional studies employing both the specific RAGE inhibitor FPS‐ZM1 and the reactive oxygen species (ROS) scavenger N‐acetylcysteine (NAC). Furthermore, correlation analyses between RAGE and NLRP3 pathway components were performed in clinical samples from T1DM patients. In the diabetic mouse model, protein expression data for RAGE and NLRP3 pathway components in cardiac tissues were examined as exploratory descriptive observations to complement the clinical findings. These findings provide evidence for a mechanism by which the AGEs/RAGE axis may contribute to T1DM cardiomyopathy and propose promising therapeutic targets for therapeutic intervention.

## 2. Methods and Materials

### 2.1. Animals

Healthy male C57BL/6 mice, 8 weeks of age, together with neonatal KM mice within postnatal Days 1–3, were used as study animals. The former were obtained from Liaoning Changsheng Biotechnology Co. Ltd. (Production License No. SYXK [Liao] 2022‐0001), whereas the latter were supplied by the Experimental Animal Center of the Second Affiliated Hospital of Harbin Medical University (Harbin, China). All animal procedures conformed to the Guidelines for the Care and Use of Laboratory Animals, the ARRIVE guidelines, and were ethically approved by the Animal Ethics Committee of Harbin Medical University (Approval No. HMUIRB2026040).

### 2.2. Drugs and Chemical Reagents

NLRP3 (Cat#ab283819) was obtained from Abcam (Cambridge, United States). Caspase‐1 P20 (Cat#22915‐1‐AP), NF‐*κ*B p65 (Cat#80979‐1‐RR), and NF‐*κ*B pp65 (Cat#82335‐1‐RR) were sourced from Proteintech (Wuhan, China). ASC (Cat#A1170), N‐GSDMD (Cat#A24476), the IL‐1*β* (Cat#RK00006), and IL‐18 (Cat# SEKM‐0019) enzyme‐linked immunosorbent assay (ELISA) kits were from ABclonal (Beijing, China). *β*‐Actin (Cat#R1207‐1) was purchased from Huabio (Harbin, China). Trypsin solution (Cat#C0201), CM‐H2DCFDA staining (Cat#S0035S), Cy3‐conjugated secondary antibody (Cat# A0521), FITC‐conjugated secondary antibody (Cat# A0568), 4 ^′^,6‐diamidino‐2‐phenylindole (DAPI) (Cat#C1005), RIPA (Cat#P0013B), protein SDS‐PAGE protein loading buffer (5X) (Cat#P0015), BCA protein concentration measurement kit (Cat#P0011), and lactate dehydrogenase (LDH) cytotoxicity assay kit (Cat#C0016) were purchased from Beyotime (Beijing, China). *α*‐Actinin (Cat#GTX11007) was from GeneTex (Texas, United States). NAC (Cat#53849ES05) was obtained from Yeasen Biotechnology (Shanghai, China). FPS‐ZM1 and MCC950 (Cat#HY‐19370 and Cat#HY‐12815), STZ (Cat#HY‐13753) were obtained from MedChemExpress (Shanghai, China). AGEs (Cat#bs‐1158P) were purchased from Bioss (Beijing, China). Isoflurane (3%) (Cat#R510‐22‐10) was from RWD Life Science (Shenzhen, China). Paraformaldehyde (Cat#P1110), hematoxylin and eosin (H&E) (Cat#G1120), and Masson′s trichrome staining (Cat# G1340) were obtained from Solarbio (Beijing, China). DMEM (Cat#C11885500BT) and FBS (Cat#A5256701) were obtained Gibco (Grand Island, New York, United States). The fluorescence‐conjugated antirabbit and antimouse IgG secondary antibodies (Cat#926‐32211/926‐32210) were acquired from LI‐COR Biosciences (Lincoln, United States). Avertin (2,2,2‐tribromoethanol) (Cat#T48402) was purchased from SIGMA (Saint Louis, United States). SYTOX green (Cat#KGE2504‐500) was from KeyGEN BioTECH (Harbin, China). PI staining kit (Cat#G023‐1‐1) was from Nanjing Jiancheng Bioengineering Institute (Nanjing, China).

### 2.3. Model Establishment, Grouping, and Administration

Animals were randomly allocated to either a control (CTL) or a T1DM group. In total, 12 mice were enrolled and allocated equally to two groups, with six mice per group. All 12 animals survived the 14‐week experimental period without any mortality, yielding a 100% survival rate in both groups. Following the experimental period, all animals underwent the complete set of assays: echocardiography, histological examination, and Western blot analysis. Prior to model induction, random allocation was stratified according to body weight and fasting blood glucose levels at baseline, aiming to balance baseline characteristics among groups. CTL mice were maintained under standard conditions. T1DM was induced via two intraperitoneal administrations using STZ at a dosage of 100 mg per kilogram of body weight after a fasting period. After a 12‐h fasting period, fasting blood glucose levels were measured in mice at 1 week after the final STZ administration. Animals whose fasting glucose remained persistently above 11.1 mmol/L were considered successful T1DM models [28].

All animals were observed for a duration of 14 weeks following the model establishment. Weekly monitoring comprised random blood glucose and body weight measurements. At 14 weeks, echocardiography was performed under isoflurane anesthesia to evaluate cardiac function. At the experimental endpoint, deep anesthesia was induced by an intraperitoneal injection of Avertin (2,2,2‐tribromoethanol, 250 mg/kg). Once the loss of pedal withdrawal reflex was confirmed, blood samples were collected from the retro‐orbital sinus via a capillary tube after gentle protrusion of the eye. The mice were then euthanized by rapid cervical dislocation. The hearts were immediately removed and flushed with ice‐cold saline. The total time from anesthetic administration to tissue collection was kept under 5 min. After centrifugation, serum obtained from the blood samples was immediately frozen at −80°C. The left ventricle was sectioned transversely. For Western blotting, the apex was frozen in liquid nitrogen and stored at −80°C; for histological examination, the base was subjected to fixation in 4% paraformaldehyde for 24 h, after which paraffin embedding was conducted.

### 2.4. Echocardiography

At study termination, cardiac function was assessed via transthoracic echocardiography. Mice were anesthetized with isoflurane inhalation delivered in oxygen; anesthesia was induced with 3% isoflurane and maintained with 1%–1.5% isoflurane at an oxygen flow rate of 0.8–1 L/min. Left ventricular ejection fraction (LVEF) and left ventricular fractional shortening (LVFS) were determined as previously described [29]. The mitral inflow E/A ratio, derived from pulsed‐wave Doppler measurements, was used to characterize diastolic performance [30].

### 2.5. Histological Staining

Myocardial tissues obtained at the experimental endpoint were fixed in 4% paraformaldehyde and then paraffin‐embedded. Histopathological assessment was carried out on sections measuring 4 *μ*m in thickness. General myocardial morphology and pathological changes were evaluated via H&E staining and Masson′s trichrome staining. Heart tissues were harvested at the end of the experimental period, fixed in 4% paraformaldehyde, and embedded in paraffin. Sections were cut at a thickness of 4 *μ*m for histological examination. In accordance with the kit instructions, H&E staining was performed to observe the general morphology and pathological changes of myocardial tissue, and Masson′s trichrome staining (Cat#G1340) was used to detect myocardial collagen deposition. We performed blinded semiquantitative scoring of myocardial injury using a previously established system [31]. To assess inflammatory cell infiltration in the myocardium, two independent investigators blinded to group allocation performed semiquantitative scoring in accordance with formerly reported methods [32]. After staining, the sections underwent observation and image acquisition via light microscopy (OLYMPUS DP80, Olympus, Tokyo, Japan).

### 2.6. Cardiomyocyte Culture and Treatment

Neonatal (1‐ to 3‐day‐old) KM mice were euthanized by decapitation under hypothermia anesthesia, and the cervical skin was disinfected with 75% ethanol before decapitation. Primary cardiomyocytes were then isolated as previously described [29]. To examine the impact of AGEs, culture medium containing 200 *μ*g/mL AGEs was applied to cardiomyocytes for 24 h [33]. In intervention experiments, cells were pretreated for 1 h with 10 *μ*M NAC [33], 20 *μ*M FPS‐ZM1 [34], or 10 *μ*M MCC950 [35] before AGEs stimulation.

### 2.7. Measurement of Intracellular ROS

A DCFDA‐based kit was employed to evaluate intracellular ROS, in accordance with the manufacturer′s instructions.

### 2.8. ELISA

Commercial ELISA kits were employed to quantify IL‐1*β* and IL‐18 in mouse plasma or cell culture supernatants. All steps were carried out in strict compliance with the manufacturer′s protocol. Absorbance was recorded on a microplate reader (ELX800, BioTek, United States), and cytokine levels were derived from standard curves.

### 2.9. Immunofluorescence Staining

Following 24 h of treatment, cells and cardiac tissue sections were fixed, permeabilized, and blocked. Immunofluorescence staining and fluorescence image acquisition were performed according to earlier reports [29]. ASC speck quantification was performed with ImageJ software, and fluorescence intensity corresponding to ASC speck formation was quantitatively analyzed [36].

### 2.10. Western Blot

Protein samples from cardiac tissues and cultured cardiomyocytes were immunoblotted using a previously reported method [29].

### 2.11. Assessment of Pyroptotic Cell Death

To assess pyroptotic cell death, membrane integrity was evaluated via SYTOX Green staining, propidium iodide (PI) incorporation, and LDH release assays. Following the indicated treatments, cardiomyocytes were stained with 5 *μ*M SYTOX Green (Cat#KGE2504‐500) for 20 min at 37°C under dark conditions. For PI incorporation, cells were stained with PI working solution (Cat#G023‐1‐1) for 8 min at 37°C under dark conditions, followed by two PBS washes to remove unbound dye. The PI working solution was obtained by diluting 5 *μ*L of the PI stock solution with 1 mL PBS, as described in the DAPI/PI kit instructions. Fluorescence micrographs were captured on an Olympus FV300 confocal microscope (Tokyo, Japan). SYTOX and PI fluorescence densities were quantified in at least three random fields per well and presented as percentages of the control group′s fluorescence density. To determine LDH release, culture supernatants were sampled after treatment. The activity of the enzyme was assessed using a commercial kit (Cat#C0016) as per the manufacturer′s recommendations, with optical density measured at 490 nm on a microplate reader (ELX800, BioTek, United States). The extent of LDH release was then calculated relative to the maximal release.

### 2.12. Bioinformatic Analysis of Public Gene Expression Data

We assessed the clinical relevance of AGER and NLRP3 inflammasome pathway activity using the publicly accessible dataset GSE123658, deposited in the NCBI GEO repository (https://www.ncbi.nlm.nih.gov/geo/). The GSE123658 dataset contains whole blood RNA‐seq data from all 39 T1DM patients available in the dataset (age range: 19–73 years; 18 females and 21 males) and 43 healthy controls. All 39 T1DM patients were included in the correlation analysis without applying any additional sample selection or exclusion criteria. Only the data from the T1DM patients were used, as the objective was to evaluate the correlation between AGER and NLRP3 inflammasome‐related genes specifically in the context of Type 1 diabetes. Detailed clinical inclusion/exclusion criteria, disease duration, comorbidities, and medication history were not available from the original study. The dataset was selected for its inclusion of expression profiles relevant to the pathway under investigation. Correlation analysis between AGER and key NLRP3 inflammasome‐related genes was conducted using the annotated metadata and preprocessed expression values from the original dataset.

### 2.13. Statistical Analysis

Statistical analyses were conducted with GraphPad Prism 9.0. Results are expressed as mean ± standard error of the mean (SEM) from at least four independent experiments, and normality was tested. Between‐group differences were assessed by Student′s *t*‐test, whereas multiple group comparisons were evaluated via one‐way analysis of variance (ANOVA). Correlations between gene expression levels in human blood samples (*n* = 39) were computed using Pearson′s correlation analysis. For the mouse heart protein data (*n* = 6), formal correlation analysis was not performed because of the limited sample size; these results are shown as descriptive observations only. Statistical significance was defined as *p* < 0.05.

## 3. Results

### 3.1. Establishment of a Mouse Model of Type 1 Diabetic Cardiomyopathy

To investigate the mechanism by which AGEs activate myocardial NLRP3 inflammasome and contribute to cardiac injury in a STZ‐induced T1DM mouse model. Diabetic induction was successfully confirmed by a significantly increase fasting blood glucose in the T1DM group (15.45 ± 0.30 mmol/L) compared with the CTL group (7.23 ± 0.38 mmol/L; *p* < 0.001; Figure 1a). Subsequent glucose monitoring further showed that random blood glucose levels remained persistently elevated in the T1DM group (approaching 31.05 ± 1.01 mmol/L), whereas those in the CTL group stayed within the normal range (< 10 mmol/L) (Figure 1b, *p* < 0.001). At baseline, both groups exhibited comparable body weight (22.39 ± 0.45 g; Figure 1c). However, the body weight trajectories of the two groups diverged markedly over time, with control mice exhibiting gradual weight gain to approximately 25.23 ± 0.71 g, whereas T1DM mice showed progressive weight loss, declining to about 20.33 ± 1.02 g by the end of the study (*p* < 0.001; Figure 1d).

**Figure 1:**
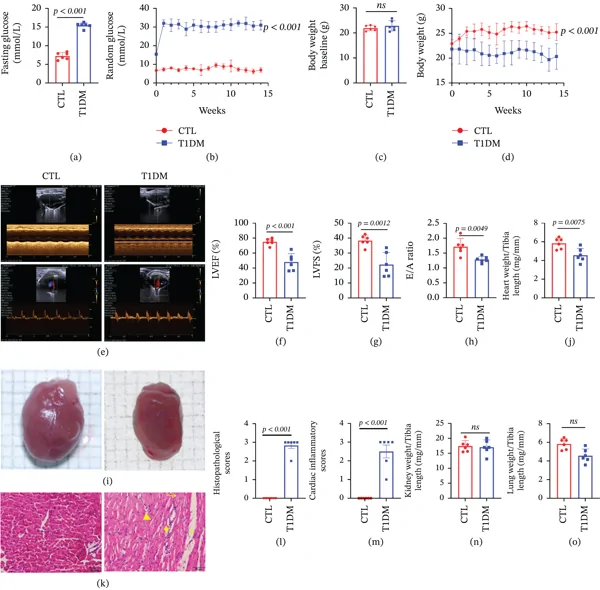
Establishment of a mouse model of Type 1 diabetic cardiomyopathy. (a) Fasting blood glucose levels measured 7 days after the last STZ injection, *n* = 6. (b) Dynamic monitoring of random blood glucose throughout the experimental period, *n* = 6. (c) Initial body weight of both groups at baseline, *n* = 6. (d) Body weight trajectories over the study duration, *n* = 6. (e) Echocardiographic assessment of cardiac function at the experimental endpoint. Top: M‐mode images; bottom: Doppler flow profiles, *n* = 6. (f–h) Quantitative analysis of cardiac systolic and diastolic function parameters measured by echocardiography, including (f) left ventricular ejection fraction (LVEF%), (g) left ventricular fractional shortening (LVFS), and (h) mitral valve inflow E/A ratio, *n* = 6. (i) Gross morphology of hearts harvested at the experimental endpoint. (j) Heart weight to tibia length (HW/TL) ratio, reflecting atrophy, *n* = 6. (k) Representative H&E‐stained sections of myocardial tissue (scale bar: 50 *μ*m). Pathological features in T1DM hearts include: disorganized and ruptured myocardial fibers (▲), cytoplasmic vacuolization (→), interstitial edema ( ^∗^), and inflammatory cell infiltration (◆), *n* = 6. (l) Semiquantitative histopathological scores based on H&E staining, *n* = 6. (m) Cardiac inflammatory score in myocardial tissue, *n* = 6. (n,o) Organ indices at the experimental endpoint. (n) Kidney weight to tibia length ratio (KW/TL). (o) Lung weight to tibia length ratio (LW/TL), *n* = 6.

Cardiac function was assessed by echocardiography at the 14‐week endpoint. Compared with the CTL group, T1DM mice exhibited markedly reduced systolic performance, as evidenced by decreased LVEF (48.13*%* ± 4.57*%* vs. 75.13*%* ± 1.82*%*; *p* < 0.001; Figure 1e,f) and LVFS (22.34*%* ± 3.31*%* vs. 38.37*%* ± 1.49*%*; *p* = 0.0013; Figure 1e,g). Diastolic dysfunction was also observed, with a notable decrease in the mitral valve inflow E/A ratio in the T1DM group (1.29 ± 0.04) relative to controls (1.72 ± 0.11; *p* = 0.0049) (Figure 1e,h). These echocardiographic abnormalities confirmed the presence of diabetic cardiomyopathy, demonstrating that both systolic and diastolic function were severely compromised in T1DM mice.

To further characterize the myocardial pathology in T1DM mice, cardiac tissue was examined macroscopically and microscopically. Gross examination of harvested hearts revealed visible atrophy in T1DM mice compared with the CTL group (Figure 1i), which was corroborated by a significantly lower heart weight‐to‐tibia length ratio (4.56 ± 0.30 vs. 5.83 ± 0.24 mg/g in controls; *p* = 0.0075; Figure 1j). Histological analysis via H&E staining further demonstrated typical myocardial injury in T1DM mice, including disordered cardiomyocyte arrangement, cytoplasmic vacuolization, interstitial edema, and inflammatory cell infiltration (Figure 1k). Semiquantitative scoring of H&E‐stained sections revealed significantly higher myocardial injury scores in T1DM mice relative to control animals (*p* < 0.001; Figure 1l). Similarly, semiquantitative analysis demonstrated increased inflammatory infiltration scores in the myocardium of T1DM mice (*p* < 0.001; Figure 1m), consistent with the chronic inflammatory nature of diabetes. To assess fibrotic remodeling, Masson′s trichrome staining was performed. Interestingly, no obvious collagen deposition was observed in the myocardium of T1DM mice compared with controls, whereas prominent fibrosis was detected in myocardial tissue from 14‐day postmyocardial infarction mice used as a positive control (Figure S1). This finding suggests that the 14‐week STZ model represents an early stage of diabetic cardiomyopathy, in which inflammatory changes precede overt fibrotic remodeling [37]. In contrast, no notable changes were detected in kidney or lung organ indices between the two groups (Figure 1n,o), indicating that the pathological effects under these experimental conditions were primarily cardiac‐specific. Together, these data substantiate that this model reproduces cardinal features of diabetic cardiomyopathy, notably cardiac atrophy and structural myocardial impairment.

### 3.2. NLRP3 Inflammasome Signaling Is Activated in Myocardial Tissue of T1DM Mice

To investigate the molecular basis of diabetic myocardial injury, we examined the activation status of the NLRP3 inflammasome pathway. Immunoblotting demonstrated markedly elevated NLRP3 levels in cardiac lysates from T1DM mice relative to CTL group (*p* = 0.0036, Figure 2a,b). In line with this, the level of cleaved caspase‐1 P20 was also markedly increased (*p* = 0.0010, Figure 2a,c). Furthermore, the active form of the downstream pyroptosis effector GSDMD (N‐GSDMD) was significantly elevated (*p* = 0.0136, Figure 2a,d), indicating caspase‐1–mediated cleavage. At the systemic level, circulating levels of IL‐1*β* and IL‐18 were substantially higher in T1DM mice (approximately 5.32‐fold and 12.98‐fold, respectively; *p* < 0.001, Figure 2e–f), which is a direct consequence of NLRP3 inflammasome activation. Morphological evidence from immunofluorescence staining confirmed enhanced ASC speck formation in T1DM myocardial tissue (*p* = 0.0014; Figure 2g,h), a hallmark of inflammasome assembly. In summary, these results indicate that T1DM induces NLRP3 inflammasome activation in cardiac tissue, leading to caspase‐1–mediated GSDMD cleavage and enhanced secretion of proinflammatory cytokines, which collectively contribute to diabetic myocardial injury.

**Figure 2:**
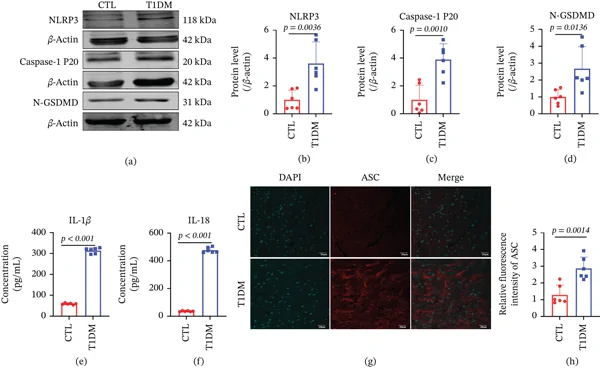
Activation of the NLRP3 inflammasome pathway in myocardial tissues of T1DM mice. (a) Representative Western blot images showing the protein expression levels of NLRP3, caspase‐1 P20, and N‐GSDMD in myocardial tissues. (b–d) Semiquantitative analysis of the protein expression levels of (b) NLRP3, (c) caspase‐1 P20, and (d) N‐GSDMD. *β*‐actin was used as a loading control, *n* = 6. (e,f) Serum concentration of (e) IL‐1*β* and (f) IL‐18 measured by ELISA, *n* = 6. (g) Representative immunofluorescence images showing ASC speck formation (red) in myocardial tissues. Nuclei were stained with DAPI (blue). Scale bar: 20 *μ*m. (h) Quantification of ASC fluorescence intensity, *n* = 6.

### 3.3. AGEs Activate the NLRP3 Inflammasome Pathway in Cultured Cardiomyocytes

Given our in vivo observation that T1DM activates NLRP3 inflammasome signaling in the heart, we next asked whether AGEs directly trigger this pathway in cultured cardiomyocytes. Cultured primary neonatal mouse cardiomyocytes were exposed to 200 *μ*g/mL AGEs. Immunoblotting revealed that AGEs markedly upregulated NLRP3 protein levels (*p* = 0.0073; Figure 3a,b) and the cleaved isoform of caspase‐1 (*p* = 0.0095; Figure 3a,c). Concurrently, the generation of the pyroptotic effector N‐GSDMD was substantially enhanced (*p* = 0.004; Figure 3a,d), indicating caspase‐1–mediated cleavage. Accordingly, secretion of IL‐1*β* and IL‐18 into the culture medium was markedly increased (*p* < 0.001; Figure 3e,f). Immunofluorescence staining further revealed increased ASC speck formation in AGEs‐treated cardiomyocytes (*p* = 0.0096; Figure 3g,h), confirming inflammasome assembly. Together, these findings establish that AGEs alone can activate the NLRP3 inflammasome in cardiomyocytes independent of other diabetic microenvironmental factors, highlighting their central contribution to NLRP3‐mediated cardiac injury in T1DM.

**Figure 3:**
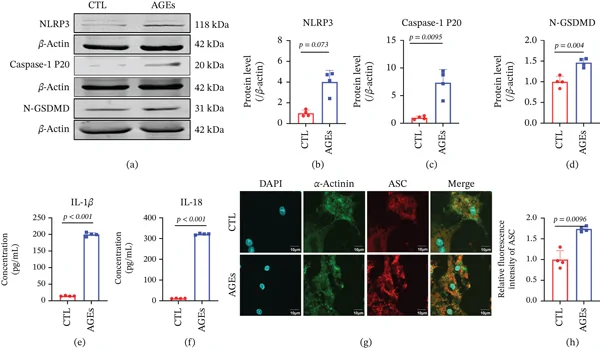
AGEs activate the NLRP3 inflammasome pathway in primary cultured cardiomyocytes. (a) Representative Western blot images showing the protein levels of NLRP3, caspase‐1 P20, and N‐GSDMD in cardiomyocytes treated with or without AGEs, *n* = 4. (b–d) Semiquantitative analysis of (b) NLRP3, (c) caspase‐1 P20, and (d) N‐GSDMD protein expression. *β*‐actin was used as a loading control, *n* = 4. (e,f) Concentration of (e) IL‐1*β* and (f) IL‐18 in the cell culture supernatant detected by ELISA, *n* = 4. (g) Representative immunofluorescence images showing ASC speck formation (red) in cardiomyocytes. Nuclei were stained with DAPI (blue). Scale bar: 10 *μ*m. (h) Quantification of ASC fluorescence intensity, *n* = 4.

### 3.4. Ages Trigger NLRP3 Inflammasome Activation in Cardiomyocytes Through RAGE‐ROS‐NF‐*κ*B Axis

Having established that AGEs activate the NLRP3 inflammasome, we sought to delineate the upstream signaling events. Given that ligation of RAGE, the principal AGE receptor, elicits intracellular oxidative stress [38], and that ROS stimulate the redox‐sensitive transcription factor NF‐*κ*B, a known positive regulator of NLRP3 expression [39, 40], we hypothesized that AGEs signal through a RAGE/ROS/NF‐*κ*B cascade. Initial experiments using the RAGE‐specific inhibitor FPS‐ZM1 demonstrated that RAGE blockade markedly attenuated the AGEs‐induced upregulation of phosphorylated NF‐*κ*B p65 (*p* < 0.001; Figure 4a,b), NLRP3 (*p* < 0.001; Figure 4a,c), caspase‐1 P20 (*p* = 0.0033; Figure 4a,d), and N‐GSDMD (*p* = 0.0058; Figure 4a,e). Consistent with the suppression of inflammasome components, FPS‐ZM1 treatment also reduced the release of IL‐1*β* (*p* < 0.001; Figure 4f) and IL‐18 (*p* < 0.001; Figure 4g), confirming that AGEs‐induced NLRP3 inflammasome activation is mediated through RAGE. We next asked whether ROS, a known downstream mediator of RAGE [38], contribute to this signaling cascade. AGEs treatment significantly increased intracellular ROS levels, as measured by DCFDA fluorescence (*p* < 0.001; Figure 4h,i). This increase was blocked by either FPS‐ZM1 (*p* < 0.001; Figure 4h,i) or ROS scavenger NAC (*p* = 0.0038; Figure 4s,t), indicating RAGE‐dependent ROS generation. Notably, scavenging ROS with NAC also suppressed the protein expression of phospho‐NF‐*κ*B p65 (*p* = 0.0072; Figure 4l,m), NLRP3 (*p* = 0.0053; Figure 4l,n), caspase‐1 P20 (*p* = 0.0016; Figure 4l,o), and N‐GSDMD (*p* = 0.0023; Figure 4l,p), as well as the secretion of IL‐1*β* (*p* < 0.001; Figure 4q) and IL‐18 (*p* = 0.0114; Figure 4r) induced by AGEs. Furthermore, both FPS‐ZM1 and NAC significantly reduced ASC speck formation in AGEs‐treated cardiomyocytes (*p* < 0.001; Figure 4j,k,u,v), supporting their inhibitory effect on inflammasome assembly. Collectively, our data support a signaling mechanism whereby AGEs drive NLRP3 inflammasome activation in cardiomyocytes through a RAGE‐initiated, ROS‐propagated cascade that ultimately leads to NF‐*κ*B–dependent transcriptional priming and inflammasome activation.

**Figure 4:**
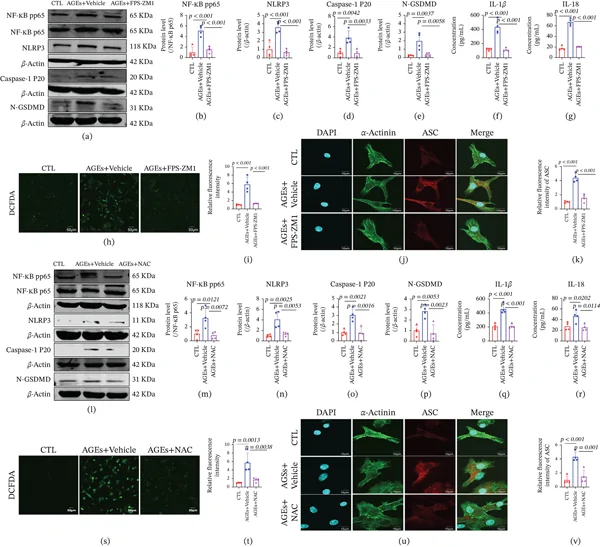
AGEs activate the NLRP3 inflammasome in cardiomyocytes via the RAGE/ROS/NF‐*κ*B signaling axis. (a–k) Primary neonatal mouse cardiomyocytes were pretreated with or without the RAGE inhibitor FPS‐ZM1 (20 *μ*M) or vehicle for 1 h, followed by stimulation with AGEs (200 *μ*g/mL) for 24 h. (a) Representative Western blots of phospho‐NF‐*κ*B p65 (pp65), total NF‐*κ*B p65, NLRP3, caspase‐1 P20, and N‐GSDMD. (b–e) Quantitative analysis of the protein levels of (b) pp65/NF‐*κ*B p65, (c) NLRP3, (d) caspase‐1 P20, and (e) N‐GSDMD, *n* = 4. (f,g) Concentrations of (f) I L‐1*β* and (g) IL‐18 in the cell culture supernatant were measured by ELISA, *n* = 4. (h,i) Intracellular ROS levels were detected using the DCFHDA fluorescent probe. (h) Representative fluorescence images. Scale bar, 50 *μ*m. (i) Quantitative analysis of ROS fluorescence intensity, *n* = 4. (j,k) ASC oligomerization was assessed by immunofluorescence. (j) Representative images showing ASC specks. Scale bar, 10 *μ*m. (k) Quantification of ASC fluorescence intensity, *n* = 4. (l–v) Cardiomyocytes were pretreated with or without the ROS scavenger NAC (10 *μ*M) or vehicle for 1 h, followed by stimulation with AGEs (200 *μ*g/mL) for 24 h. (l) Representative immunoblots of the indicated proteins. (m–p) Quantitative analysis of (m) pp65/NF‐*κ*B p65, (n) NLRP3, (o) caspase‐1 P20, and (p) N‐GSDMD, *n* = 4. (q,r) ELISA analysis of (q) IL‐1*β* and (r) IL‐18 levels in the supernatant, *n* = 4. (s,t) Intracellular ROS levels were measured as above. (s) Representative images. Scale bar, 50 *μ*m. (T) Fluorescence quantification, *n* = 4. (u,v) Assessment of ASC speck formation. (u) Representative images. Scale bar, 10 *μ*m. (v) Quantification of ASC fluorescence intensity, *n* = 4.

### 3.5. Clinical Correlation of the AGEs/RAGE Pathway With NLRP3 Inflammasome Activation and In Vivo Validation

To test the clinical translatability of our mechanistic observations, we first examined publicly available transcriptomic data from the GSE123658 dataset. Whole blood RNA‐seq data from 39 T1DM patients (age range: 19–73 years; 18 females and 21 males) were used to examine the relationship between AGER and NLRP3‐related transcripts. Demographic details of these patients are listed in Table S1. Analysis of the GEO dataset demonstrated a marked positive association of AGER (encoding RAGE) with IL‐1*β* (*r* = 0.889, *p* < 0.001; Figure 5a) and IL‐18 (*r* = 0.634, *p* < 0.001; Figure 5b) in circulating blood, implying a possible connection between RAGE signaling and inflammasome activation in T1DM patients. We next examined whether this association is reflected at the protein level in diabetic heart tissue. In T1DM mouse hearts, RAGE protein levels were markedly elevated in the myocardium relative to control mice (*p* = 0.0015; Figure 5c,d). Individual‐level expression data for RAGE, NLRP3, and N‐GSDMD in T1DM mouse heart are shown in Figure 5e,f. Given the small cohort (*n* = 6), these data are presented as exploratory descriptive observations, without formal statistical testing. Collectively, the human transcriptomic data and the experimental animal findings indicate that engagement of the AGEs/RAGE axis may correlate with NLRP3 inflammasome‐associated inflammation in the Type 1 diabetic heart. The in vitro mechanistic data, which demonstrated a causal relationship between AGEs stimulation and NLRP3 inflammasome activation in cardiomyocytes, provide the primary causal evidence for the proposed pathway, whereas the in vivo observations offer complementary descriptive support.

**Figure 5:**
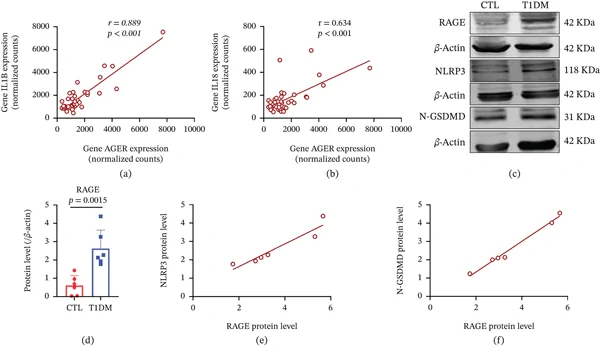
Clinical association and in vivo characterization of the AGEs/RAGE/NLRP3 inflammasome axis in T1DM cardiomyopathy. (a,b) Analysis of publicly available transcriptomic data (GEO Accession: GSE123658) from whole blood samples of T1DM patients and healthy controls. Scatter plots show a significant positive correlation between AGER (RAGE) mRNA expression and transcript levels of (a) IL‐1*β* and (b) IL‐18. Pearson′s correlation coefficients (*r*) and *p* values are indicated in the figure, *n* = 39. Each point represents an individual subject. (c–f) Protein analysis in myocardial tissues from control and T1DM mice. (c) Representative Western blots showing protein levels of RAGE, NLRP3, and N‐GSDMD. (d) Quantitative analysis of RAGE protein expression normalized to *β*‐actin, *n* = 6. (e,f) Scatter plots showing individual‐level expression of RAGE protein versus (e) NLRP3 and (f) N‐GSDMD in T1DM mouse hearts. Each point represents an individual mouse. A linear trend line is shown to illustrate the overall pattern of the data, *n* = 6.

### 3.6. NLRP3 Inflammasome Is Essential for AGEs‐Induced Pyroptosis in Cardiomyocytes

To directly determine whether NLRP3 inflammasome activation is indispensable for AGEs‐induced inflammatory responses and pyroptotic cell death in cardiomyocytes, we employed the specific NLRP3 inhibitor MCC950. Primary neonatal mouse cardiomyocytes were pretreated with MCC950 (10 *μ*M) prior to AGEs stimulation. Immunoblotting revealed that MCC950 treatment markedly downregulated NLRP3 protein expression (*p* < 0.001; Figure 6a,b). Accordingly, the levels of cleaved caspase‐1 P20 (*p* = 0.015; Figure 6a,c) and N‐GSDMD (*p* = 0.004; Figure 6a,d) were also markedly decreased, indicating effective blockade of inflammasome activation and subsequent GSDMD cleavage. Consistent with these changes, MCC950 treatment significantly suppressed the release of IL‐1*β* and IL‐18 into the culture medium (*p* < 0.001; Figure 6e,f).

**Figure 6:**
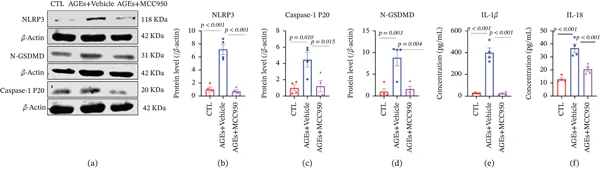
NLRP3 is required for AGEs‐induced inflammatory responses in cardiomyocytes. Primary neonatal mouse cardiomyocytes were pretreated with or without the NLRP3 inhibitor MCC950 (10 *μ*M) or vehicle for 1 h, followed by stimulation with AGEs (200 *μ*g/mL) for 24 h. (a) Representative Western blots of NLRP3, caspase‐1 p20, and N‐GSDMD. (b–d) Quantitative analysis of the protein levels of (b) NLRP3, (c) caspase‐1 P20, and (d) N‐GSDMD, *n* = 4. (e,f) Concentrations of (e) IL‐1*β* and (f) IL‐18 in the cell culture supernatant were measured by ELISA, *n* = 4.

To further assess whether AGEs trigger pyroptotic cell death in cardiomyocytes through the RAGE/ROS/NLRP3 pathway, we performed membrane integrity assays. Cardiomyocytes were treated with AGEs in the presence or absence of FPS‐ZM1, NAC, or MCC950. Compared with the control cohort, AGEs treatment substantially increased the number of SYTOX‐positive cells, indicating loss of membrane integrity (*p* < 0.001; Figure 7a,b). This increase was significantly attenuated by FPS‐ZM1, NAC, and MCC950 (*p* < 0.001 for all groups; Figure 7a,b). Consistent with these findings, PI uptake, another well‐established marker of membrane permeabilization, was significantly elevated in AGEs‐treated cardiomyocytes and was reduced by all three inhibitors (*p* < 0.001; Figure 7c,d). Furthermore, LDH release into the culture medium was significantly increased in AGEs‐treated cardiomyocytes and was attenuated by all three inhibitors (*p* < 0.001; Figure 7e). Collectively, these data demonstrate that AGEs induce pyroptotic cell death in cardiomyocytes through the RAGE/ROS/NLRP3/caspase‐1/GSDMD axis, and that targeting any component of this pathway can effectively attenuate pyroptosis.

**Figure 7:**
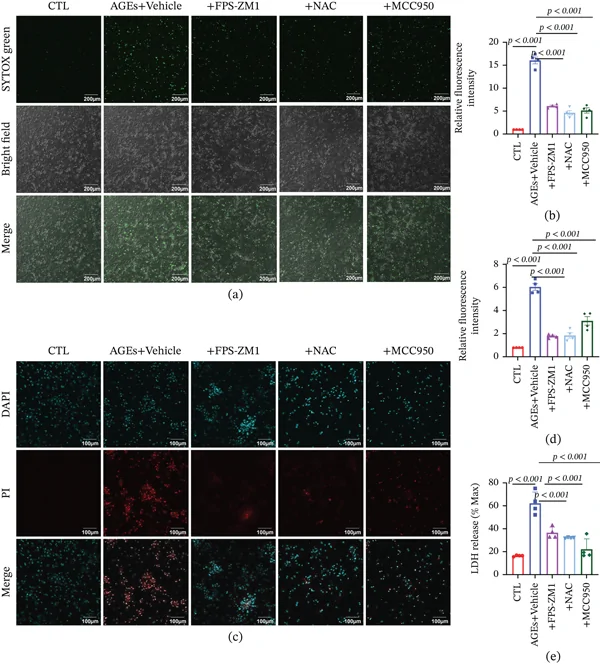
AGEs induce pyroptotic cell death in cardiomyocytes through the RAGE/ROS/NLRP3 pathway. Primary neonatal mouse cardiomyocytes were pretreated with or without FPS‐ZM1 (20 *μ*M), NAC (10 *μ*M), MCC950 (10 *μ*M), or vehicle for 1 h, followed by stimulation with AGEs (200 *μ*g/mL) for 24 h. (a) Representative fluorescence images of SYTOX green staining. Scale bar, 200 *μ*m. (b) Quantitative analysis of SYTOX‐positive cells, *n* = 4. (c) Representative fluorescence images of propidium iodide (PI) uptake. Scale bar, 100 *μ*m. (d) Quantitative analysis of PI‐positive cells, *n* = 4. (e) Lactate dehydrogenase (LDH) release in the cell culture supernatant was measured, *n* = 4.

## 4. Discussion

This study provides evidence that the AGEs/RAGE/ROS/NF‐*κ*B signaling axis may function as a pathway involved in NLRP3 inflammasome activation and myocardial inflammation in Type 1 diabetic cardiomyopathy. Using a multilevel approach combining in vivo, in vitro, and clinical analyses, we found that STZ‐induced diabetic mice exhibited cardiac dysfunction accompanied by NLRP3 inflammasome activation, that AGE stimulation in cardiomyocytes triggered NLRP3 inflammasome assembly and pyroptosis via a RAGE/ROS/NF‐*κ*B‐dependent mechanism, and that RAGE expression correlated positively with circulating IL‐1*β* and IL‐18 in T1DM patients. In the diabetic mouse model, cardiac RAGE and NLRP3 protein levels were coupregulated, consistent with the in vitro findings. Together, these findings suggest a metabolic‐inflammatory pathway that may contribute to the pathogenesis of T1DM cardiomyopathy.

Of note, the current work did not directly measure myocardial or circulating AGE concentrations. However, the accumulation of AGEs in the diabetic myocardium has been well documented. In postmortem cardiac tissue from patients with diabetes, increased AGE deposition has been detected by immunohistochemistry, and these levels correlate with the extent of myocardial fibrosis and diastolic impairment [41, 42]. In addition, in rodent models of Type 1 diabetes induced by STZ, elevated AGE levels in cardiac tissue have been consistently reported [43–45]. These findings support the rationale for the presence of AGEs in the diabetic heart and for employing the STZ model in the current study.

The AGEs/RAGE axis has been extensively characterized in diabetic microvascular disease, notably nephropathy and retinopathy [46–50], whereas NLRP3 inflammasome activation is recognized as a contributor to several cardiovascular pathologies, especially diabetic cardiomyopathy [19, 50]. The ROS/NF‐*κ*B signaling cascade is a well‐established mechanism linking metabolic stress to inflammatory responses in multiple cell types. However, the role of the AGEs/RAGE pathway in myocardial inflammation in Type 1 diabetes, and in particular its connection to NLRP3 inflammasome activation, has been less clear. The present study addresses this gap by showing that the AGEs/RAGE pathway and NLRP3 inflammasome can be coactivated in cardiomyocytes under conditions relevant to T1DM. By combining clinical data, animal models, and in vitro experiments, we found that RAGE expression was positively associated with serum IL‐18 and IL‐1*β* concentrations in T1DM patients. Furthermore, in the diabetic mouse heart, individual‐level protein expression data for RAGE, NLRP3, and N‐GSDMD showed a consistent pattern of coexpression, which is presented as exploratory descriptive observations.

The STZ‐induced C57BL/6 mouse model of T1DM faithfully recapitulates early‐stage human diabetic cardiomyopathy. At 14 weeks postinduction, these animals exhibited key pathological features including reduced heart weight and impaired systolic and diastolic function, without significant alterations in indices of other major organs such as the lungs or kidneys [51]. This phenotype mirrors the early clinical phase of T1DM cardiomyopathy, which is characterized by primary myocardial injury prior to the onset of systemic circulatory failure [52]. The 14‐week duration in mice corresponds to the prolonged subclinical phase in T1DM patients, which can span 25–30 years before progressing to overt heart failure [53], providing a relevant time window for studying the initiating mechanisms of the disease. An additional advantage of this model is the pronounced cardiac‐specific susceptibility to diabetic metabolic stress exhibited by the C57BL/6 strain, with minimal concurrent injury to the lungs or kidneys within the same timeframe [51]. This organ selectivity allows for a clearer investigation of the direct effects of hyperglycemia on the myocardium, largely independent of secondary damage from other organ systems. Furthermore, the STZ‐induced model, which directly induces hyperglycemia through *β*‐cell toxicity, is particularly suitable for investigating metabolic insults such as AGEs accumulation, as it isolates hyperglycemia‐driven myocardial pathology from confounding immune‐mediated components of *β*‐cell destruction [54]. Collectively, the model provides a robust platform for dissecting the initial molecular events in T1DM cardiomyopathy.

To investigate the relationship between the AGEs/RAGE axis and NLRP3 inflammasome regulation, we performed in vitro experiments using primary neonatal mouse cardiomyocytes. AGE stimulation promoted NLRP3 inflammasome assembly and subsequent activation, resulting in pyroptosis, suggesting that AGEs accumulating under hyperglycemic conditions can initiate an autologous inflammatory response in cardiomyocytes independent of systemic immunity. Mechanistically, AGE engagement of RAGE triggered a substantial rise in intracellular ROS, which functioned as signaling molecules upstream of NF‐*κ*B, whose activation was associated with the transcriptional upregulation of NLRP3 and subsequent inflammasome assembly. The role of this pathway was further supported by intervention studies: Both the RAGE‐specific inhibitor FPS‐ZM1 and the antioxidant NAC disrupted this signaling cascade and reduced NLRP3 activation. These findings suggest that the AGEs/RAGE/ROS/NF‐*κ*B signaling axis can regulate NLRP3 inflammasome activation in cardiomyocytes, providing evidence for a mechanistic link between sustained metabolic stress and the activation of innate immune pathways in the Type 1 diabetic heart.

We acknowledge that the in vivo data in this study are primarily descriptive and demonstrate associations rather than causation. The correlative observations between RAGE expression and NLRP3 pathway activation in diabetic mouse hearts, together with the clinical correlations in T1DM patients, establish the pathophysiological relevance of this axis. However, the causal relationship at the whole‐organ level is supported by the in vitro experiments, in which direct AGEs stimulation of cardiomyocytes triggered NLRP3 inflammasome activation and pyroptosis in a RAGE‐ and ROS‐dependent manner, and pharmacological inhibition of RAGE with FPS‐ZM1 or ROS scavenging with NAC abrogated these effects. These in vitro findings provide direct causal evidence at the cellular level that mechanistically explain the correlative in vivo observations. The consistency of findings across clinical, in vivo, and in vitro levels offers complementary support for the proposed mechanism.

There are several limitations to the present study that warrant consideration. First, the correlation analysis of RAGE, NLRP3, and N‐GSDMD in mouse cardiac tissues was based on a relatively small number of animals (*n* = 6); these results are reported as exploratory descriptive findings and should not be overinterpreted. The in vivo experiments were designed to establish associations rather than causation, and whereas the in vitro experiments provided mechanistic insights at the cellular level, in vivo intervention studies using pharmacological inhibitors (e.g., FPS‐ZM1, NAC, and NLRP3 inhibitors) or genetic models (e.g., cardiomyocyte‐specific RAGE or NLRP3 knockout) are needed to establish causation at the whole‐organ level. Second, the STZ model, though widely used, has inherent limitations. STZ exerts direct cytotoxic effects on multiple tissues, including the heart, independent of hyperglycemia [55], which may contribute to the observed cardiac abnormalities. Alternative diabetic models such as the Akita [56] or OVE26 mouse [57] would help distinguish hyperglycemia‐driven effects from STZ‐related toxicity. Third, several methodological considerations regarding the in vitro experiments warrant attention. The use of commercially prepared AGEs may not fully capture the heterogeneity of endogenously formed AGEs under prolonged metabolic stress, which could influence the physiological relevance of the findings. Additionally, the present study did not directly measure myocardial or circulating AGEs concentrations, nor did it directly assess RAGE receptor activation. However, the accumulation of AGEs in the Type 1 diabetic heart has been well documented in the literature [43–45], and the functional necessity of RAGE in the AGEs‐induced signaling cascade was established using the specific pharmacological inhibitor FPS‐ZM1. Furthermore, neonatal mouse cardiomyocytes, though a well‐established mode [58–64], differ from adult cardiomyocytes in metabolism, proliferative capacity, and responses to stress stimuli. Future studies using adult cardiomyocytes or human iPSC‐derived cardiomyocytes would be valuable to confirm generalizability. This study also focused on cardiomyocyte‐intrinsic inflammatory responses; the contributions of noncardiomyocyte populations, including fibroblasts or immune cells, to AGEs‐mediated inflammation remain to be explored. Fourth, the GSE123658 dataset used for clinical correlation analysis provides only limited demographic information (age and sex), and detailed clinical characteristics such as disease duration, comorbidities, and medication history were unavailable. Future studies with comprehensive clinical phenotyping are warranted. Finally, several translational challenges should be considered. For FPS‐ZM1, preclinical efficacy has been demonstrated in diabetic cardiomyopathy models [65–67], but human safety data are lacking. For NAC, a systematic review protocol has suggested potential benefits against hyperglycemia‐induced cardiac oxidative damage [68], yet clinical evidence for diabetic cardiovascular complications remains limited. Targeting the NLRP3 inflammasome faces challenges due to incomplete understanding of its activation mechanisms and the difficulty of developing selective inhibitors [37, 69].

Future investigations should be aimed at clarifying cell type–specific mechanisms within the heart and exploring the therapeutic potential of targeting the RAGE/ROS/NF‐*κ*B/NLRP3 pathway in more integrated disease models. Specifically, in vivo studies employing cardiomyocyte‐specific RAGE or NLRP3 knockout mice, or pharmacological interventions with FPS‐ZM1, NAC, or selective NLRP3 inhibitors in diabetic animal models are warranted to establish causation at the whole‐organ level and to assess the therapeutic potential of this pathway. Additionally, future studies should investigate the contributions of nonmyocyte populations, including fibroblasts and immune cells, to AGEs‐mediated myocardial inflammation and should seek to validate the clinical associations identified here using cohorts with more comprehensive phenotyping. Notwithstanding these limitations, the current results enhance our understanding of potential nonimmune‐related pathological mechanisms that may contribute to cardiomyopathy in Type 1 diabetes and may provide a basis for further exploration of the AGEs/RAGE/ROS/NF‐*κ*B/NLRP3 cascade as a potential therapeutic target.

## 5. Conclusion

In summary, this study suggests a potential role for the AGEs/RAGE/ROS/NF‐*κ*B signaling pathway in the pathogenesis of Type 1 diabetic cardiomyopathy. The present findings are consistent with the possibility that hyperglycemia‐induced AGEs may promote myocardial inflammation through NLRP3 inflammasome activation and pyroptosis in cardiomyocytes, a process that appears to be mechanistically dependent on RAGE‐mediated ROS generation and NF‐*κ*B signaling. The consistency of findings across clinical data, animal studies, and in vitro assays supports the pathophysiological relevance of this pathway. In addition, pharmacological inhibition of RAGE or ROS was found to attenuate this cascade, suggesting potential therapeutic relevance. Taken together, these findings contribute to a conceptual framework for understanding metabolic‐inflammatory crosstalk in the Type 1 diabetic heart and may provide a basis for further exploration of the AGEs/RAGE/ROS/NF‐*κ*B/NLRP3 cascade as a target for therapeutic intervention in T1DM cardiomyopathy.

## Author Contributions

Xiaoqun Li: writing—original draft preparation, investigation, data curation, visualization, formal analysis. Shujie Chen and Ke Wang: investigation, data curation, formal analysis. Ke Xu: resources. Yannan Wang: investigation, data curation. Zechao Zhang: investigation, data curation. Xue Liu: funding acquisition, supervision, writing—review and editing.

## Funding

This study was supported by the National Natural Science Foundation of China (10.13039/501100001809; 81700220); Chunyan Team Programme of Heilongjiang Province (CYQN24043); and the Postdoctoral Startup Foundation of Heilongjiang Province (21042230016).

## Disclosure

All authors have read and approved the final version of the manuscript. Xue Liu had full access to all of the data in this study and takes complete responsibility for the integrity of the data and the accuracy of the data analysis.

## Ethics Statement

This study was reviewed and approved by the Ethics Committee of Harbin Medical University (Approval No. HMUIRB2026040).

## Conflicts of Interest

The authors declare no conflicts of interest.

## Supporting Information

Additional supporting information can be found online in the Supporting Information section.

## Supporting information


**Supporting Information 1** Figure S1: Assessment of myocardial fibrosis by Masson′s trichrome staining. Representative images of Masson′s trichrome‐stained cardiac sections from CTL, T1DM, and 14‐day postmyocardial infarction (MI) mice (positive control). Collagen fibers are stained blue. No obvious collagen deposition was observed in CTL or T1DM myocardium, whereas prominent fibrosis was detected in the infarcted area of MI hearts. Scale bar: 50 *μ*m. *n* = 6.

**Supporting Information 2** Table S1: Demographic and clinical characteristics of T1DM patients from the GSE123658 dataset.

**Supporting Information 3** File S1: The ARRIVE guidelines 2.0: author checklist.

## Data Availability

The data that support the findings of this study are available from the corresponding author upon reasonable request.
