# Supplementary material for: The AGEs/RAGE Axis Contributes to Myocardial Cell Death in Type 1 Diabetic Cardiomyopathy via the ROS/NF‐κB/NLRP3 Inflammasome Pathway
Source: J Diabetes Res. 2026 Oct 23;2026:6438008. doi: 10.1155/jdr/6438008 (PMC13691523; doi:10.1155/jdr/6438008)
Supplement: Supplementary file 1 [file JDR-2026-6438008-s003.pdf]

NOTE: Please save this file locally before filling in the table, DO NOT work on the file within your internet browser as changes will not be saved. Adobe Acrobat Reader (available free here) is recommended for completion.

The ARRIVE guidelines 2.0: author checklist

The ARRIVE Essential 10

These items are the basic minimum to include in a manuscript. Without this information, readers and reviewers cannot assess the reliability of the findings.

Item		Recommendation	Section/line number, or reason for not reporting
**Study design**	1	For each experiment, provide brief details of study design including:<br>a. The groups being compared, including control groups. If no control group has been used, the rationale should be stated.<br>b. The experimental unit (e.g. a single animal, litter, or cage of animals).	groups (n=6 per group) and a cell study (n=4 per condition) comprising five parts: Part 1 (CTL vs. AGEs), Part 2 (CTL, AGEs+Vehicle, AGEs+FPS-ZM1), Part 3 (CTL, AGEs+Vehicle, AGEs+NAC), Part 4 (CTL, AGEs+Vehicle, AGEs+MCC950), and Part 5 (CTL, AGEs+Vehicle, AGEs+FPS?ZM1, AGEs+NAC, AGEs+MCC950).<br>omyocytes derived from 20 neonatal mice were pooled to generate a single sample. Four independent cage-based biological replicates were included for each experimental condition.
**Sample size**	2	a. Specify the exact number of experimental units allocated to each group, and the total number in each experiment. Also indicate the total number of animals used.<br>b. Explain how the sample size was decided. Provide details of any *a priori* sample size calculation, if done.	4 independent biological replicates derived from 4 separate cages, and cardiomyocytes pooled from each cage were aliquoted to different treatment groups within the same subpart to avoid redundant animal consumption.<br>animal experiment, the calculated required sample size was 2–4 per group, and 6 per group were actually used. For the cell experiment, the calculated required sample size was 2–4 per group, and 4 per group were actually used.
**Inclusion and exclusion criteria**	3	a. Describe any criteria used for including and excluding animals (or experimental units) during the experiment, and data points during the analysis. Specify if these criteria were established *a priori*. If no criteria were set, state this explicitly.<br>b. For each experimental group, report any animals, experimental units or data points not included in the analysis and explain why. If there were no exclusions, state so.<br>c. For each analysis, report the exact value of *n* in each experimental group.	9 ± 0.45 g) and a fasting blood glucose level < 11.1 mmol/L prior to model induction were included. For the in vitro study, neonatal KM mice aged 1–3 days old were used. No animals or cell samples were excluded from the final analysis.<br>No animals, experimental units or data points were excluded from analysis in any experimental group. For in vivo mouse experiments, n=6 biological replicates per group were fully included for statisti<br>Sample sizes: n = 6 biological replicates per group for in vivo experiments; n = 4 biological replicates per experimental group for in vitro cellular assays.
**Randomisation**	4	a. State whether randomisation was used to allocate experimental units to control and treatment groups. If done, provide the method used to generate the randomisation sequence.<br>b. Describe the strategy used to minimise potential confounders such as the order of treatments and measurements, or animal/cage location. If confounders were not controlled, state this explicitly.	Random allocation was adopted to assign experimental mice to control (CTL) and T1DM model groups. Stratified random grouping was performed based on baseline body weight and fasting blood glucose levels to balance baseline characteristics across groups. The randomisation sequence was generated via simple random number g<br>Mice housed in identical standard cages, evenly distributed cage positions across rack; blood collection, echocardiography and tissue dissection performed in randomised order to avoid measurement bias.
**Blinding**	5	Describe who was aware of the group allocation at the different stages of the experiment (during the allocation, the conduct of the experiment, the outcome assessment, and the data analysis).	animal anesthesia procedures, as well as Western blot and immunofluorescence assays for both animal and cell samples, with full awareness of group allocation. Outcome assessment & Data analysis stage: The two researchers from Researcher Team 3 completed all outcome indicator evaluations under full blinding, with no access to group allocation information.
**Outcome measures**	6	a. Clearly define all outcome measures assessed (e.g. cell death, molecular markers, or behavioural changes).<br>b. For hypothesis-testing studies, specify the primary outcome measure, i.e. the outcome measure that was used to determine the sample size.	of the animal experiment. Additionally, the effects of AGEs treatment and inhibitor interventions on cell death were assessed by SYTOX staining, propidium iodide (PI) staining, and lactate dehydrogenase (LDH) release assay.<br>the sample size was not determined by a power analysis based on a specific endpoint. Post hoc power analysis was performed for all experimental comparisons and confirmed adequate statistical power.
**Statistical methods**	7	a. Provide details of the statistical methods used for each analysis, including software used.<br>b. Describe any methods used to assess whether the data met the assumptions of the statistical approach, and what was done if the assumptions were not met.	All statistical analyses performed using GraphPad Prism 9.0. Two-group comparisons: unpaired Student's t-test; multiple-group comparisons: one-way ANOVA; human blood gene correlation: Pearson correlation analysis.<br>Statistical analyses were conducted with GraphPad Prism 9.0. Results are expressed as mean ± standard error of the mean (SEM) from at least four independent experiments, and normality was tested. Between-group differences were assessed by
**Experimental animals**	8	a. Provide species-appropriate details of the animals used, including species, strain and substrain, sex, age or developmental stage, and, if relevant, weight.<br>b. Provide further relevant information on the provenance of animals, health/immune status, genetic modification status, genotype, and any previous procedures.	ao) 2022-0001).Neonatal KM mice at developmental stage of postnatal day 1–3 were obtained from the Experimental Animal Center of the Second Affiliated Hospital of Harbin Medical University, Harbin, China.<br>Healthy male C57BL/6 mice, aged 8 weeks, were obtained from Liaoning Changsheng Biotechnology Co., Ltd. (production license No.: SYXK [Liao] 2022-0001)). Neonatal KM mice (1-3 days old) were obtained from the Experimental Animal Center of t
**Experimental procedures**	9	For each experimental group, including controls, describe the procedures in enough detail to allow others to replicate them, including:<br>a. What was done, how it was done and what was used.<br>b. When and how often.<br>c. Where (including detail of any acclimatisation periods).<br>d. Why (provide rationale for procedures).	ination, heart tissue collection, primary cardiomyocyte isolation, and AGE/inhibitor treatment are all elaborated in the experimental methods to ensure the reproducibility of the experiment.<br>cted synchronously in both control group and STZ-induced diabetic model mice at the same weekly fixed time point to screen hypoglycemia and infectious complications.<br>t/12 h dark cycle. Mice had ad libitum access to standard laboratory chow and sterile drinking water, and no experimental manipulations or interventions were performed during this period.<br>ty, consistent with the reported mechanism that the AGEs/RAGE axis mediates myocardial cell death in type 1 diabetic cardiomyopathy through the ROS/NF-κB/NLRP3 inflammasome pathway.
**Results**	10	For each experiment conducted, including independent replications, report:<br>a. Summary/descriptive statistics for each experimental group, with a measure of variability where applicable (e.g. mean and SD, or median and range).<br>b. If applicable, the effect size with a confidence interval.	protein expression levels, cytokine concentrations, fluorescence intensity and cell death percentage of all groups. All data passed normality test, thus parametric statistical methods were applied accordingly.<br>sons (Student's t-test, one-way ANOVA), Pearson correlation analysis reported correlation coefficient r with 95% CIs. Mouse cardiac protein data with limited sample size only provided descriptive statistics without formal effect size calculation.

The Recommended Set

These items complement the Essential 10 and add important context to the study. Reporting the items in both sets represents best practice.

Item		Recommendation	Section/line number, or reason for not reporting
**Abstract**	11	Provide an accurate summary of the research objectives, animal species, strain and sex, key methods, principal findings, and study conclusions.	ury and is a promising therapeutic target.
**Background**	12	a. Include sufficient scientific background to understand the rationale and context for the study, and explain the experimental approach.	Sufficient literature background about T1DM cardiomyopathy, AGEs/RAGE and NLRP3 inflammasome was provided.
		b. Explain how the animal species and model used address the scientific objectives and, where appropriate, the relevance to human biology.	al whole blood samples from T1DM patients to further validate the in vivo animal experimental findings, which demonstrates the biological reference value of this animal model for research on human diabetic myocardial injury.
**Objectives**	13	Clearly describe the research question, research objectives and, where appropriate, specific hypotheses being tested.	ptosis to promote T1DM myocardial injury.
**Ethical statement**	14	Provide the name of the ethical review committee or equivalent that has approved the use of animals in this study, and any relevant licence or protocol numbers (if applicable). If ethical approval was not sought or granted, provide a justification.	All animal procedures were conducted in accordance with the Guideline for the Care and Use of Laboratory Animals and received approval from the Animal Ethical Committees of Harbin Medical University (Approval NO. HMUIRB2026040).
**Housing and husbandry**	15	Provide details of housing and husbandry conditions, including any environmental enrichment.	g without extra environmental enrichment.
**Animal care and monitoring**	16	a. Describe any interventions or steps taken in the experimental protocols to reduce pain, suffering and distress.	Avertin isoflurane were used for anesthesia to reduce animal pain during sampling and ultrasound detection.
		b. Report any expected or unexpected adverse events.	No unexpected death or severe adverse reactions occurred in all mice within 14 weeks.
		c. Describe the humane endpoints established for the study, the signs that were monitored and the frequency of monitoring. If the study did not have humane endpoints, state this.	sumption, fur condition, faeces and body weight. Diabetic mice received additional daily blood glucose testing. Animals displaying overt pain, severe weakness or moribund signs were humanely euthanized promptly.
**Interpretation/ scientific implications**	17	a. Interpret the results, taking into account the study objectives and hypotheses, current theory and other relevant studies in the literature.	Results were interpreted combined with preset hypothesis and existing published literatures.
		b. Comment on the study limitations including potential sources of bias, limitations of the animal model, and imprecision associated with the results.	All study limitations were elaborated, including small mouse sample size, inherent defects of STZ model, difference between neonatal and adult cardiomyocytes, lack of in vivo gene knockout verification.
**Generalisability/ translation**	18	Comment on whether, and how, the findings of this study are likely to generalise to other species or experimental conditions, including any relevance to human biology (where appropriate).	yopathy. The findings derived from the STZ?induced T1DM C57BL/6 mouse model and primary neonatal cardiomyocytes are partially generalizable to other rodent T1DM models with AGE accumulation. However, caution should be taken when extrapolating these results to non?rodent species, other diabetic etiologies, or different experimental conditions.
**Protocol registration**	19	Provide a statement indicating whether a protocol (including the research question, key design features, and analysis plan) was prepared before the study, and if and where this protocol was registered.	No pre-study experimental protocol registered on public platform; complete experimental design and analysis plan were fully described in manuscript Methods section.
**Data access**	20	Provide a statement describing if and where study data are available.	esponding author upon reasonable request.
**Declaration of interests**	21	a. Declare any potential conflicts of interest, including financial and non-financial. If none exist, this should be stated.	All authors declare no financial or non-financial conflicts of interest.
		b. List all funding sources (including grant identifier) and the role of the funder(s) in the design, analysis and reporting of the study.	ovince, and the Chunyan Team Programme of Heilongjiang Province (Grant No. CYQN24043). The funders had no role in study design, data collection and analysis, decision to publish, or preparation of the manuscript.
