# Supplementary material for: The AGEs/RAGE Axis Contributes to Myocardial Cell Death in Type 1 Diabetic Cardiomyopathy via the ROS/NF‐κB/NLRP3 Inflammasome Pathway
Source: J Diabetes Res. 2026 Oct 23;2026:6438008. doi: 10.1155/jdr/6438008 (PMC13691523; doi:10.1155/jdr/6438008)
Supplement: Supplementary file 2 [file JDR-2026-6438008-s001.docx]

**Supplementary Figure 1. Assessment of myocardial fibrosis by Masson’ s trichrome staining.** Representative images of Masson’ s trichrome-stained cardiac sections from CTL, T1DM, and 14-week post-myocardial infarction (MI) mice (positive control). Collagen fibers are stained blue. No obvious collagen deposition was observed in CTL or T1DM myocardium, whereas prominent fibrosis was detected in the infarcted area of MI hearts. Scale bar: 50 μm. n = 6.
