# Supplementary material for: The AGEs/RAGE Axis Contributes to Myocardial Cell Death in Type 1 Diabetic Cardiomyopathy via the ROS/NF‐κB/NLRP3 Inflammasome Pathway
Source: J Diabetes Res. 2026 Oct 23;2026:6438008. doi: 10.1155/jdr/6438008 (PMC13691523; doi:10.1155/jdr/6438008)
Supplement: Supplementary file 3 [file JDR-2026-6438008-s002.docx]

**Supplementary Table 1. Demographic and clinical characteristics of T1DM patients from the GSE123658 dataset.**

| Characteristic | Value |
| --- | --- |
| Number of patients | 39 |
| Sex (female/male) | 18 / 21 |
| Sample type | Whole blood |
| Data type | RNA-seq |
| Disease type | Type 1 diabetes mellitus |
| Detailed inclusion criteria | Not reported in original dataset |
| Exclusion criteria | Not reported in original dataset |
| Disease duration | Not reported in original dataset |
| Comorbidities | Not reported in original dataset |
| Medication history | Not reported in original dataset |
